# Electrochemical etching of metals and minerals using ultrasound in deep eutectic solvents^☆^

**DOI:** 10.1016/j.ultsonch.2025.107403

**Published:** 2025-05-27

**Authors:** Philip Hunt, Jennifer M. Hartley, Muwafaq A. Rabeea, Andrew P. Abbott, Christopher E. Elgar

**Affiliations:** a School of Chemistry, University of Leicester, Leicester LE1 7RH, United Kingdom; b Department of Applied Chemistry, College of Applied Sciences-Hit, University Of Anbar, Iraq

**Keywords:** Ultrasound, Deep eutectic solvent, Electropolishing, Mineral dissolution, Alloy

## Abstract

This study compares the anodic dissolution of two iron-based alloys, mild steel and an FeNdB super magnet, with that of a mineral, chalcopyrite (CuFeS_2_), in a deep eutectic solvent formed from choline chloride and ethylene glycol under silent and ultrasonic conditions. These three materials have different granular morphologies and heterogeneities. The aim was to show how a combination of ultrasound and electrochemistry can lead to different etching mechanisms through increased mass transport by the mechanical action of bubbles at the electrode surface. Linear sweep and cyclic voltammetries showed that both alloys passivated on the anodic sweep. Using ultrasound significantly decreases passivation, but does not fully prevent it as was previously proposed. Partial passivation produces enhanced pitting and anisotropic etching of all grains. Where the alloy grains are heterogeneous, as is the case with FeNdB, anisotropic etching can lead to surface fragmentation under certain conditions, with partial grains being dislodged from the surface without dissolution. In this case, selective dissolution of the Nd-rich phase was observed. Cyclic voltammetry of chalcopyrite showed several anodic redox processes under silent conditions, but the use of ultrasound led to a linear current–voltage response with a roughly 15-fold increase in the current. Electrochemical etching occurs around grain boundaries, making the material more prone to further ultrasonic fragmentation. This led to a rapid separation of the copper-rich phase from the largely silica-based gangue phase. The surface was inert under just insonation, demonstrating the importance of the anisotropic electrochemical etching process. This approach could be used for the purification of minerals.

## Graphical abstract

**Figure:**
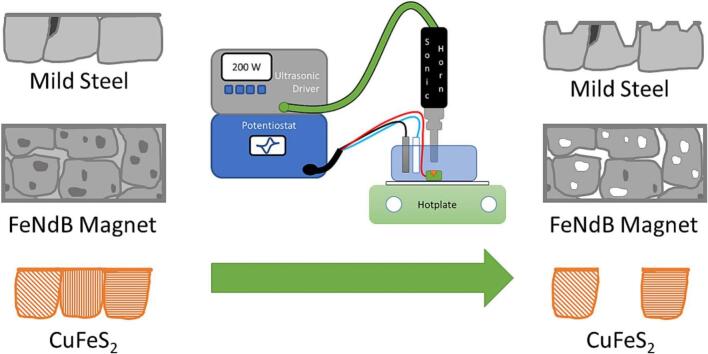

## 1 Introduction

Electrochemical etching of metals is well known and is routinely carried out for surface patterning, micromachining, surface finishing and levelling. In many cases, metal dissolution is limited by the formation of passivating films which slows the migration of metal ions and ligands to obtain a controlled surface corrosion. This is most commonly used in the process of electropolishing, where macro-defects are removed from a surface to leave a bright surface which can passivate evenly. The electropolishing of stainless steel has been relatively well studied in a range of solvents including concentrated mineral acids [1,2], and in non-aqueous systems such as ionic liquids or deep eutectic solvents (DES).[[3], [4], [5]] Other metals such as copper, cobalt, nickel, and titanium have also been successfully electropolished using a range of solvent systems. [[6], [7], [8], [9], [10], [11]] In general, electropolished surfaces are achieved through the mechanism of controlled corrosion, involving the formation of a viscous, potentially supersaturated, layer at the metal/solution interface which protects recessed regions of the electrode surface from being etched as fast as the protruding regions.[2,12] The rate limiting step to metal dissolution is thought to be related to either diffusion of the dissolved metal species away (either as ‘free’ ions or metal complexes) from the electrode before they can form localised saturated layers at the electrode surface, or diffusion of ligands towards the electrode surface. Passive films are also used to enhance corrosion resistance, as in the case of stainless steel. However, these passivated metals can prevent electropolishing of metals with particularly inert metal oxides.

The electrochemical etching of minerals has also been demonstrated, mainly as an avenue towards the extraction of metals from chemically resistant minerals, such as metal sulfides. A major issue with extracting metals from sulfide minerals is that the mineral surface passivates via the formation of metal-poor sulfur/polysulfide-rich layers, regardless of whether chemical or electrochemical methods are used.[[13], [14], [15]] Most studies in non-aqueous media have used chemical etching and these show relatively slow etch rates and passivation is still prevalent. A few studies have investigated electrochemical etching of minerals in DESs, and while etch rates were faster than chemical etch rates due to the larger driving force of a higher over potential, rates were limited by passivation. Minerals including copper-, iron-, arsenic-, and lead-based sulfides were shown to electrochemically leach in DESs. [[16], [17], [18]] In the case of chalcopyrite (CuFeS_2_) it was shown that both metals were etched from the mineral and careful choice of the DES could enable selective recovery of the copper over iron. In unstirred conditions, depletion of the metal ions left a sulfur-rich material which passivated the electrode.

One of the major differences presented by using ionic liquids or deep eutectics in metal or minerals processing is their generally much higher viscosity compared to aqueous solvents,[19,20] hence significantly impacting mass transports rates for both metal complexes and ligands. This mass transport issue can be overcome either by diluting the solvent, e.g. with water, [[21], [22], [23], [24]] or by using some form of forced convection, e.g. stirring or ultrasound [25,26].

Cavitation has three main effects in sonoelectrochemical experiments. Firstly, as the sound wave propagates through the liquid, some energy is absorbed by the surroundings, causing an increase in fluid mixing. This is known as acoustic streaming.[27] Secondly, the cavitation waves cause localised mixing of the solvent, and increasing mass transport rates between the bulk solvent and the double layer, either bringing more active species to the surface or removing passivating species. Finally, if cavitation occurs close to a surface, the bubble will collapse asymmetrically. Asymmetric collapse leads to a phenomena known as jetting, where the imploding bubble shoots a forceful jet at the surface.[28] This can lead to the bulk removal of material from the surface for softer materials or the disruption of the electrical double layer (EDL), causing an increase in the rate of mass transport [26].

Traditional hydrometallurgy processes that have benefitted from application of ultrasound are numerous, for example ores processing[[29], [30], [31]], magnet recycling[32] and critical metal extraction from spent batteries[[33], [34], [35]]. Sonoelectrochemical dissolution however is used more rarely in the literature. The use of ultrasound during electropolishing will increase the current density. It is also able to aid in the removal of surface protrusions.[36] Li et al explored the use of sonoelectrodissolution of chalcopyrite oxidation in sulfuric acid, improving dissolution rates by 45 % under insonated control over silent conditions.[37] The superimposition of ultrasound during anodic etching of aluminium has been used by Kang et al as a method of limiting the growth of oxide films and increasing the surface area by preferential pit corrosion growth for aluminium electrolytic capacitors, which require a large surface area [38].

Under the presence of ultrasound, it was found that metals were electrochemically etched in concentrated ionic media at remarkably similar rates, regardless of oxidation state or solution species. It was proposed that migration of the dissolved metal species across the double layer was the rate limiting factor towards dissolution.[39] The only metals which did not electrochemically etch tended to be those which form particularly resilient passivation layers, such as titanium and platinum.

**Fig. 1:**
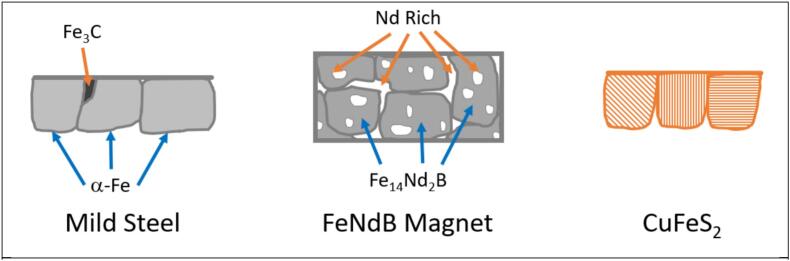
The different phase behaviour of the 3 different iron alloys tested in this work.

In this study the effects of ultrasound have been tested on three different iron-based alloys, chosen for their different phase behaviours (Fig. 1). Mild steel exhibits grains of α-Fe with smaller interstitial grains of FeC_3_. FeNdB alloys consist of iron rich, Fe_14_Nd_2_B grains, surrounded by a Nd rich (Nd_4_Fe) phase and a B rich phase (NdFe_4_B_4_) at the grain boundaries, both of which are typically more active sites.[40,41] Chalcopyrite mineral samples consist of large grains of different crystal orientations, likely interspersed with other phases, such as quartz, along some grain boundaries.[42] The electrochemical etching has been compared between the alloys showing that the mechanism of dissolution under silent conditions depends upon the phase behaviour of the material being tested, with the rate of sonoelectrodissolution also being dependent upon this mechanism.

## 2 Experimental

### 2.1 Solvent preparation

The chemicals used in this study were used in their supplied condition, without further purification. The deep eutectic solvent was prepared from a 1:2 M ratio of choline chloride (ChCl) (Sigma Aldrich, >98 %) and ethylene glycol (EG) (Fisher Scientific, 98 %). The components were stirred together at 70 °C using a hotplate until a colourless homogeneous liquid was formed. The prepared solvent was transferred from the hotplate and stored in a 50 °C oven in sealed storage bottles and used within 3 weeks of preparation.

### 2.2 Electrochemistry

A Metrohm Autolab PGSTAT204 potentiostat operating the corresponding Nova 2.1 software was used to perform the Cyclic voltammetry (CV) and anodic linear sweep voltammetry (LSV) experiments, using a 3-electrode system (**Fig. S1**). The working electrode is made of either mild steel plates, chalcopyrite chunks or NdFeB magnet chunks set into resin. Chalcopyrite electrodes were produced from small, high purity, chunks (<10 mm thick) of mineral sourced from Norway & Wheal Penrose Cornwall through supplier; Richard Tayler Minerals, UK. The chalcopyrite was connected to insulated copper wire and embedded in MetPrep Variset resin. The demagnetized permanent magnet sample was kindly provided by the University of Birmingham. The weight percentages of iron (Fe), neodymium (Nd), dysprosium (Dy), and praseodymium (Pr) in the used NdFeB were determined to be 67.8 %, 24.2 %, 3.7 %, and 3.5 % respectively by ICP-MS analysis [43].

The counter electrode was an iridium oxide-coated titanium mesh, and the reference electrode was 0.09 mol dm^–3^ AgCl/Ag in ChCl:2EG. The working electrodes were polished sequentially on 800, 1200 and 3200 grit sandpaper (Metprep, UK). The LSVs were recorded at a scan rate of 10 mV s^–1^, at a temperature of 50 °C using a thermocouple hotplate.

For insonated LSV experiments, a commercial ultrasonic horn (Branson Sonics, 1.25DCXa20V) was used. The system had a cylinder sonotrode of 20 mm diameter, operating at 20 kHz, with a power variable up to 1250 W (maximum power intensity of 398 W cm^−2^). The distance from horn-surface-to-electrode was fixed at 4 mm, with a consistent power density of 132 W cm^–2^ at the probe tip. Silent- and Sono- electrochemical etching experiments of the chalcopyrite were carried out using a EX355R 2-wire power supply.

### 2.3 Imaging

For optical imaging, a Zeta 20 Optical Profiler and the associated Zeta3D software version 1.8.5, maintaining a constant 5x − 100x magnification was used. The number of images within a stitch varied from 2x2 to 8x8 to fully encapsulate surface effects. Inbuilt functions within the Zeta3D software were used to calculate the roughness parameters of images obtained.

Surface morphology and elemental were measured with a Quanta FEG 650 scanning electron microscope (SEM) (ThermoFisher Scientific, USA) equipped with an XMax energy dispersive X-ray spectroscopy (EDX) (Oxford Instruments, UK), and an accelerating voltage of 20 kV at the University of Leicester’s Advanced Microscopy Facility.

## 3 Results & discussion

**Fig. 2:**
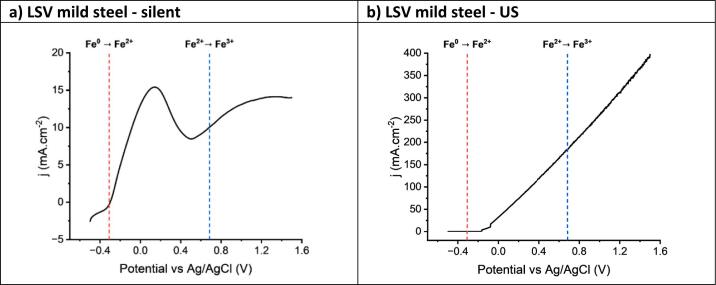
Linear sweep voltammetry of a mild steel working electrode in ChCl:2EG under a) silent, and b) ultrasonic conditions. The sweep rate was 10 mV s^–1^, and the temperature was 50 °C. The counter electrode was an iridium oxide-coated titanium mesh, and the reference was 0.09 mol dm^−3^ AgCl/Ag in ChCl:2EG. The applied ultrasound was 132 W cm^–2^. The dashed lines are used to highlight the location of the formal redox onset potentials of the known redox processes present.

Previously the electrochemical etching of copper and nickel were studied under an ultrasonic horn. Relatively uniform etching was observed for both metals. Under silent conditions the nickel showed pitting and it was assumed that the ultrasound combined with high chloride concentration removed the passive film and prevented pitting. In this study mild steel (iron with ca. 0.30 wt% carbon) was used as a substrate because it is a heterogeneous alloy composed of grains of a solid solution of C in Fe with grains of Fe_3_C. Aqueous systems enable passive layers of iron oxides and hydroxides to form. In this study, the etching medium was a high chloride solvent formed of 1 mol choline chloride (ChCl) and 2 mol ethylene glycol (EG). In this medium there is also the possibility for Fe-glycol solids to form.[44,45] Voltammetry under silent conditions (Fig. 2**a**) shows that mild steel has multiple oxidative features but it behaves extensively in the same way as pure iron.[45] The initial oxidation at ca. –0.3 V vs the 0.09 mol dm^–3^ AgCl/Ag in ChCl:2EG reference electrode can be directly linked to the oxidation of Fe^0^ to Fe^2+^. The oxidation of Fe^2+^ to Fe^3+^ should commence around ca. + 1.0 V after the Fe^2+/0^ redox couple (Fig. 2**a**).[46] Under ultrasound, a constant increase in current is observed instead, at a gradient of 239 mA V^–1^ cm^–2^. The linear current–potential response of the ultrasound assisted LSVs has previously been investigated for copper, silver, aluminium, cobalt, and nickel. Gradients of 200–600 mA V^–1^ cm^–2^ were obtained under an applied ultrasound power of 132 W cm^–2^, and it was proposed that this linear behaviour was due to a change to migration control rather than a diffusion limited process that would be controlling the system in silent conditions [26,39].

The area under the voltammogram is directly related to total charge passed during the anodic dissolution process. The total charge density passed for the silent system is 2.1C cm^–2^, whereas the ultrasound system is 30.9C cm^–2^; this is an increase in total charge passed and mass dissolved by approximately 15 times. The large increase in current density caused by the introduction of acoustic streaming from the ultrasonic horn improves mass transport rates to and from the electrode’s surface, as well as cavitation shock waves disrupting the double layer that would promptly form in the silent systems. This in turn removes iron species from the electrode-solution interface ensuring that the local concentration of the iron species does not reach super-saturation and passivate the electrode surface.

**Fig. 3:**
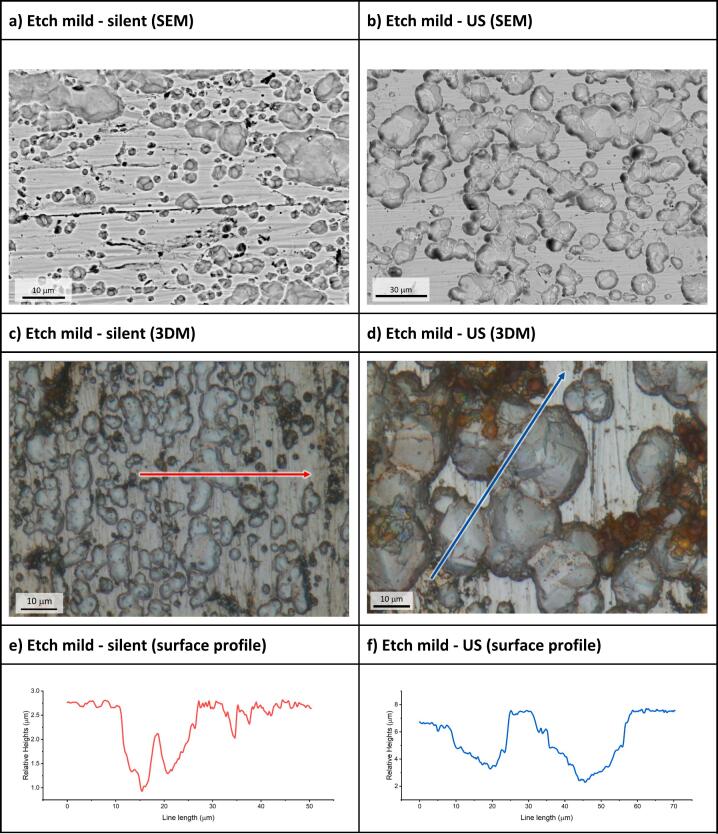
The surface of mild steel plates after chronoamperometry experiments in ChCl:2EG at 50 °C for 180 s. Plates were held at + 1.5 V vs 0.09 mol dm^–3^ AgCl/Ag in ChCl:2EG. a) and b) show silent and insonated plates respectively, taken with backscatter mode SEM (BSE-SEM). c) and d) show the same plates from optical tomography microscopy, with the line indicating where height cross section was measured. e) and f) show the surface profile along the lines. The applied ultrasound was 132 W cm^–2^, and the horn tip was located 4 mm above the steel plate.

The electro-etching of mild steel in ChCl:2EG at 50 °C with a constant applied potential of + 1.5 V measured against 0.09 mol dm^–3^ AgCl/Ag in ChCl:2EG for 3 min can be seen in Fig. 3. Under silent conditions it can be seen that mild steel etches, to produce a rough surface with significant pitting, producing an average roughness across the surface of Ra = 0.35 (±0.06) µm, a change from the pre-exposed surface of Ra = 0.1 (±0.01) µm. This arises from a partial passive film on the mild steel surface. Electrochemical etching of mild steel under the ultrasonic horn produces a well-defined circular area which is clearly darker under the horn (**Fig. S2**) than the surrounding area where slower mass transport occurred. The darker area is etched more deeply but it is quite clear that the pitting is even more pronounced. Fig. 3**b** shows the same experiment under ultrasonic conditions and it is evident that less of the surface passivated under the horn, but it is also clear that a passive layer is still present and hence the linear current–voltage response in Fig. 2**b** does not mean that the surface does not passivate as previously thought.[39] Rather, it means that the current is not simply diffusion controlled.

Fig. 3**a** shows that a few grain boundaries can be observed at the base of the pits, but Fig. 3**c** shows that the base of these pits are not very wide or deep and the bases are not well defined. Fig. 3**b and 3d** show SEM and 3D microscopy images of the electrochemically etched surface under the ultrasonic horn. Here the pits are much wider and deeper, and the bases of the pits show clear grain boundaries with angular interfaces suggesting that the etching is anisotropic. The x-cut profile through the surface shows holes which are ca. 10 to 20 µm across, which are in keeping with the grain size of most mild steel samples. The 3D optical image of Fig. 3**d** can be seen in **Fig. S3**. Fig. 3**e** shows an etch depth of approximately 1 µm, whereas Fig. 2**a** corresponds to approximately 0.5 µm. This difference can be reconciled because only about half of the surface is etched. The etch depth under ultrasound in Fig. 3**f** is approximately 5 µm, which would account for the larger charge passed, given that a larger fraction of the surface is etched. The ability to anisotropically etch surfaces is particularly useful with imaging high performance superalloys [47].

**Fig. 4:**
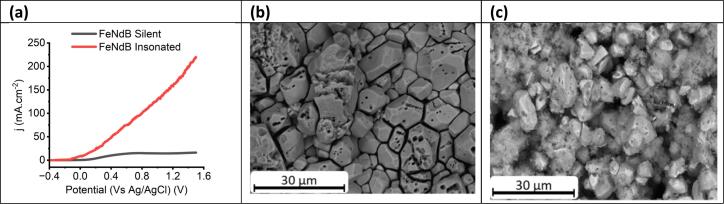
a) Linear sweep voltammograms of FeNdB working electrodes in ChCl:2EG, in silent and under ultrasonic conditions. The scan was recorded at a scan rate of 30 mV s^−1^, and with a 0.1 mol dm^−3^ AgCl/Ag in ChCl:2EG reference electrode. **b)** SEM image for the NdFeB after US-electrochemical in ChCl:2EG. **c)** SEM image of the precipitate phase after US-electrochemical etching of NdFeB in ChCl:2EG.

In contrast to the dissolution of homogeneous single metal samples, the dissolution of a simple heterogeneous mild steel alloy shows that passivation is present even under ultrasonic conditions and fast etch rates leads to anisotropic etching. This shows that grain composition is extremely important. This idea can be extended by considering FeNdB, which is an alloy where there are compositional differences within a single grain. Specifically, there are Nd-rich phases which occur as islands and around grain boundaries. Fig. 4**a** shows the voltammetry of a FeNdB supermagnet under silent and insonated conditions. As with Fig. 2, similar responses are observed i.e. passivation under silent conditions and a roughly linear current–voltage response under ultrasound.

The etched surface under ultrasonic conditions, Fig. 4**b**, shows severe pitting in the middle of the grains and around the grain boundaries, consistent with preferential dissolution of the Nd-rich phases. Bulk electrochemical dissolution results in a magnetic, metallic residue at the base of the cell. This was isolated and imaged (Fig. 4**c**) and it can be seen that the particles look similar to the surface from which they have been dislodged. This leads to partial fragmentation of the surface, aided by ultrasonic jetting, but with dissolution of the valuable Nd phase. This example confirms the results for mild steel in demonstrating anisotropic etching through the joint action of ultrasound and electrochemistry, but in this case, preferential solubility of grain edges enables granular fragmentation of the surface.

It was recently shown that metal oxides could be electrodissolved through oxidation of the oxide to form the superoxide and this is analogous to transpassive corrosion of the metal oxides during electropolishing. Many of the important minerals which are now being mined are sulfides, and here the oxidation is more complicated due to the complex redox chemistry of sulfur and particularly the polysulfide anion.[48] For example, during chemical processing, minerals such as chalcopyrite and pyrite form passivation layers rich in insoluble sulfur species. There are numerous species which can be produced, but one example is:

$${2CuFeS}_{2}-{5e}^{-}\to {\mathrm{Cu}}^{2+}+{\mathrm{Fe}}^{3+}+{\mathrm{CuFeS}}_{4}$$

**Fig. 5:**
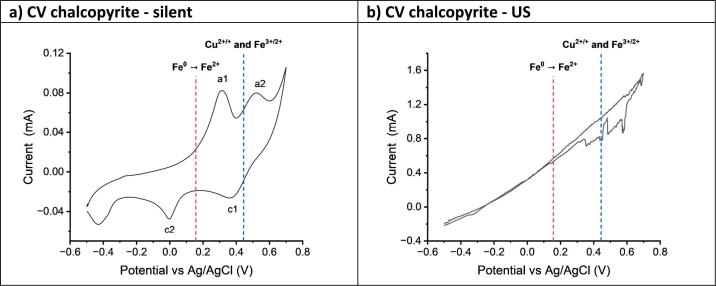
Cyclic voltammetry of a chalcopyrite working electrode in ChCl:2EG under a) silent, and b) ultrasonic conditions. The sweep rate was 10 mV s^–1^, and the temperature was 50 °C. The counter electrode was a platinum flag, and the reference was 0.09 mol dm^–3^ AgCl/Ag in ChCl:2EG. Note that the chalcopyrite electrode was ca. 3 mm thick, with an exposed surface area of ca. 18.10 (+/- 0.5) mm^2^. The applied ultrasound was 132 W cm^–2^. The dashed lines are used to highlight the location of the formal redox couples of the known redox processes present.

In order to process these ores at more economic rates, the passivation layer must be removed, either by ensuring the passivating species are soluble, or via immediate removal as it forms. Since improved mass transport from the application of ultrasound has shown the ability to prevent passivation layers in mild steel through the prevention of passivation layer formation, it should provide the same benefit to sulfide-containing minerals, such as pyrite or chalcopyrite. The dissolution rates of the mineral can be further enhanced via anodic oxidation of the sulfide into different sulfur-containing species, such as polysulfides, sulfites or sulfates, which often have improved solubility in solution. The presence of chloride ions has also been shown to help solubilise the different metallic species, as well as sulphur-containing species into solution.[49,50] The DES ChCl:2EG has a chloride concentration of ca. 3.8 mol kg^–1^, and has been shown to be suitable for the electrochemical leaching and recovery of copper from chalcopyrite, [16] and other chalcogenide-based minerals.[18,51] Voltammetry of chalcopyrite shows that under silent conditions, there are three observable redox processes seen (Fig. 5**a**). Anggara et al[16] assigned the redox process at ca. + 0.45 V vs 0.09 mol dm^–3^ AgCl/Ag in ChCl:2EG to the overlapping Cu^2+/+^ and Fe^3+/2+^ redox couples, the redox couple at ca. + 0.15 V to a sulfur/sulfide redox couple, and a reduction process below –0.3 V was proposed to be related to the electrodeposition of Cu or Fe metal. In the present work, the overlapping Cu^2+/+^ and Fe^3+/2+^ redox couples (a2, c1) are present at E = (E_pa_ + E_pc_)/2 = (0.52 V + 0.36 V)/2 = 0.44 V, whereas the sulfur/sulfide (a1, c2) redox couple is present at E = (E_pa_ + E_pc_)/2 = 0.16 V. Small variations between the literature and present work are due to slightly different concentrations of the reference electrode solution and natural variations in mineral composition.

Under ultrasonic conditions, a straight line was observed between applied potential and current density, similarly to the pure metals and the alloys in Fig. 2**b and 3b**. None of the previously observed redox processes were observed, indicating that none of them are reaching a state of diffusion control, as seen for the pure metal systems. While the electrochemically active surface area of the chalcopyrite electrode was not known exactly, due to it being made of a naturally occurring mineral, it was estimated to be ca. 18.10 +/- 0.5 mm^2^. This equates to a line with a gradient of between 9.0 and 9.5 mA V^–1^ cm^–2^. This is much smaller than for copper or iron, suggesting that a different rate limiting factor must be considered. In a metal, the mass transport was proposed to be limited due to migration across the electrical double layer.[39] As chalcopyrite is a semiconducting mineral, the conductivity of the mineral may be this rate limiting step instead. It is notable is that the current starts at a reducing current at the start of the voltammogram, indicating that the ultrasound is potentially enhancing a reductive process in the chalcopyrite as well; possibly Cu^+/0^ or Fe^2+/0^, depending on the oxidation states of the elements in the mineral. It should also be noted that the potential at which the current switches from reducing to oxidising is more cathodic under ultrasonic conditions, possibly due to a decrease in the apparent activation energy of the oxidation processes.[52] Much like seen during the electrochemistry of the steels, the application of ultrasound during the CV of chalcopyrite increases total charge passed by a factor of 17 times.

**Fig. 6:**
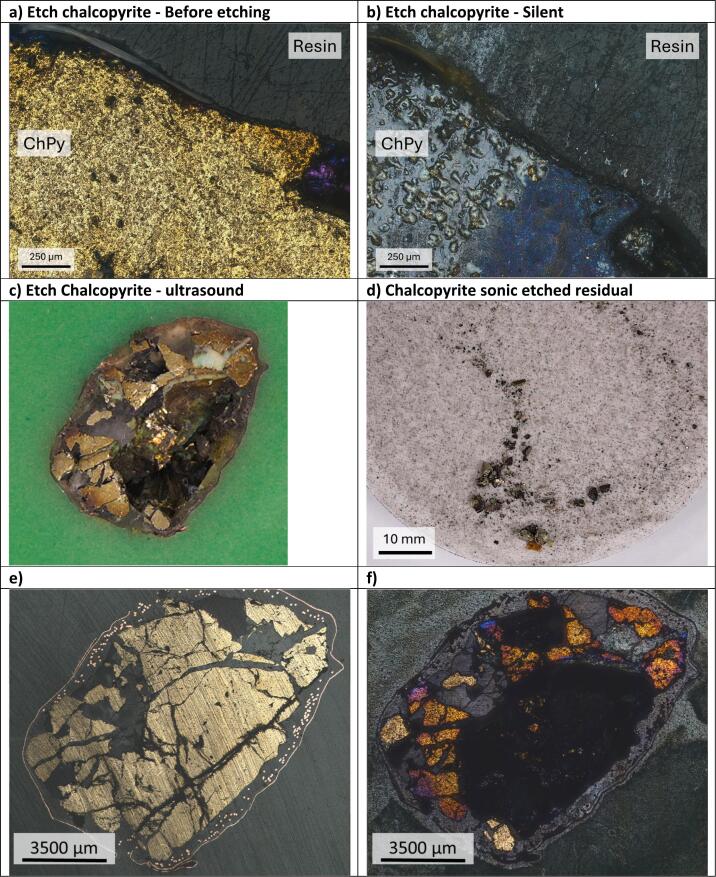
3DM images of chalcopyrite etched under a) before etching, b) Silent etching conditions, and c) ultrasonic conditions. d) residue remaining after electrochemical etching under an ultrasonic horn. Electrochemical etching was carried out at a constant applied current density of ca. 5 mA cm^−2^, for 45 min silent and 2 min insonated, at a constant temperature of 50 °C. The applied ultrasound was 132 W cm^–2^. e) and f) Before and after optical microscopy images of the chalcopyrite sample for the sonoelectrochemical dissolution experiment showing the removal of different areas of chalcopyrite, while the quartz areas remain intact.

Chalcopyrite etching under silent conditions showed a blue layer on the surface (Before Fig. 6**a**, after Fig. 6**b**). This is thought to be composed of a mostly poly-sulfur species, and of at least ca. 5–10 μm thick, since negligible copper or iron signals were visible within the same regions upon EDX analysis. The etch rate is calculated to be 0.55 ± 0.17 μm min^–1^. Note that this is a natural mineral, with different crystal faces and grains visible on the surface, so this is an average number taken from 3 different sites across the surface.

Under ultrasound conditions, the blue layer is absent and the mineral is more rapidly dissolved than under silent conditions (Fig. 6**c**). The mineral sample behaves in a similar manner to that observed for the FeNdB sample shown in Fig. 4. After only 2 min of electrochemical etching with ultrasound about half of the sample had totally fragmented, leaving large shards of the mineral at the base of the cell, as can be seen in Fig. 6**d**. While the amount of dislodged material varies, the maximum depth was recorded at around 3 mm (**Fig. S5b**). The material removed appears to have been limited by the amount of chalcopyrite in the block, as the base of the removed area is exposed copper tape used to form electrical conductivity during the block construction. Areas of the bulk mineral where no fragmentation occurred (**Fig. S5a)**, had an increase in etch rate to ca. 1.25 ± 0.42 μm min^–1^, equivalent to roughly 2.25 times increase in etching rate over silent conditions. The areas where parts of the mineral haven't been dislodged, there is a change in colour indicating a possible variety of passivating species such as metal poly-sulfides, oxides or chlorides (Before Fig. 6**e**, after Fig. 6**f**).

Significant electrochemical anisotropic etching of minerals has previously been reported under silent conditions,[17] although fragmentation was not observed in this case. When the above experiment was repeated without an applied voltage but with ultrasound, no fragmentation of the sample was observed. This suggests that the combination of electrochemical etching and ultrasound promotes etching around the edge of each grain, possibly exposing natural weaknesses in the mineral which enables bubble nucleation to dislodge shards of material (**Fig. S6**).

EDX analysis of the shards in Fig. 6**d** showed only signals for Fe, Cu and S, with no Si signals present, showing that the method only works for electrochemically active phases (**Figs. S7 and S8**). This could be a potential method for the purification of minerals from non-conducting gangue phases. The ability to electrochemically process minerals has previously been demonstrated by a process known as paint casting where a paste of minerals is confined in a DES close to an electrode surface. One method by which this could be scaled up using the information from this study would be to use a sonic horn as the current collector in an electrochemical cell and confine a paste of the mineral close to the electrode using a porous plastic tube or mesh, similar to that shown previously for silent conditions.[16].

## 4 Conclusions

The anodic electrochemical dissolution of mild steel in a DES was investigated with and without ultrasound to determine how enhanced mass transport affects the dissolution rate and surface morphology of the substrate. The etching rate of mild steel was approximately 15 times faster under forced mass transport conditions. Passivation of the steel surface was still observed with ultrasound, although it was significantly decreased compared to silent conditions. Under ultrasonic conditions, pitting was observed due to removal of the passivation layer and the effect of ultrasonic jetting. There was also evidence of anisotropic etching of the different crystal faces.

Anisotropic etching was also observed for the electrochemical dissolution of FeNdB. The Nd-rich parts of the grain were preferentially oxidised around the grain boundaries. This coupled with ultrasonic jetting led to fragmentation of the surface. Chalcopyrite was found to behave in a similar electrochemical manner to the mild steel and FeNdB samples, i.e. linear sweep voltammetry under ultrasonic conditions displayed a linear current density–voltage response. This was ascribed to a change in the dissolution mechanism from diffusion limited processes to migration limited. Anisotropic electrochemical etching of chalcopyrite was observed in DESs and this, combined with ultrasonic agitation, led to fragmentation of the surface releasing shards of pure chalcopyrite and enabling separation from the gangue silica phases.

## CRediT authorship contribution statement

**Philip Hunt:** Writing – review & editing, Writing – original draft, Methodology, Investigation, Data curation. **Jennifer M. Hartley:** Writing – review & editing, Writing – original draft, Supervision, Investigation, Formal analysis. **Muwafaq A. Rabeea:** Investigation, Data curation. **Andrew P. Abbott:** Writing – review & editing, Writing – original draft, Visualization, Supervision, Project administration, Methodology, Funding acquisition, Formal analysis, Conceptualization. **Christopher E. Elgar:** Writing – review & editing, Writing – original draft, Visualization, Validation, Supervision, Project administration, Methodology, Investigation, Formal analysis, Data curation, Conceptualization.

## Declaration of competing interest

The authors declare that they have no known competing financial interests or personal relationships that could have appeared to influence the work reported in this paper.

## References

[1] Mohan S., Kanagaraj D., Sindhuja R., Vijayalakshmi S., Renganathan N.G. (2001). Electropolishing of Stainless Steel—a Review. Trans. IMF.

[2] Han W., Fang F. (2019). Fundamental aspects and recent developments in electropolishing. Int J Mach Tool Manu.

[3] Abbott A.P., Capper G., Swain B.G., Wheeler D.A. (2005). Electropolishing of stainless steel in an ionic liquid. Trans. IMF.

[4] Abbott A.P., Capper G., McKenzie K.J., Ryder K.S. (2006). Voltammetric and impedance studies of the electropolishing of type 316 stainless steel in a choline chloride based ionic liquid. Electrochim. Acta.

[5] Abbott A.P., Capper G., McKenzie K.J., Glidle A., Ryder K.S. (2006). Electropolishing of stainless steels in a choline chloride based ionic liquid: an electrochemical study with surface characterisation using SEM and atomic force microscopy. Phys. Chem. Chem. Phys..

[6] Acquesta A., Monetta T. (2021). The Electropolishing of Additively Manufactured Parts in Titanium: State of the Art. Adv. Eng. Mater..

[7] Loftis J.D., Abdel-Fattah T.M. (2019). Nanoscale electropolishing of high-purity nickel with an ionic liquid. Int J Miner Metall Mater.

[8] Lebedeva O., Kultin D., Zakharov A., Kustov L. (2021). Advantages of Electrochemical Polishing of Metals and Alloys in Ionic Liquids. Metals.

[9] Karim W.O., Abbott A.P., Cihangir S., Ryder K.S. (2018). Electropolishing of nickel and cobalt in deep eutectic solvents. Trans. IMF.

[10] Kityk A.A., Protsenko V.S., Danilov F.I., Kun O.V., Korniy S.A. (2019). Electropolishing of aluminium in a deep eutectic solvent. Surf. Coat. Technol..

[11] Kityk A., Pavlik V., Hnatko M. (2023). Green electropolishing using choline chloride-based deep eutectic solvents: A review. J. Mol. Liq..

[12] Landolt D. (1987). Fundamental aspects of electropolishing. Electrochim. Acta.

[13] Mikhlin Y. (2000). Reactivity of pyrrhotite surfaces: An electrochemical study. Phys. Chem. Chem. Phys..

[14] Debernardi G., Carlesi C. (2013). Chemical-Electrochemical Approaches to the Study Passivation of Chalcopyrite. Miner. Process. Extr. Metall. Rev..

[15] Chanturiya V.A., Krasavtseva E.A., Makarov D.V. (2022). Electrochemistry of Sulfides: Process and Environmental Aspects. Sustainability.

[16] Anggara S., Bevan F., Harris R.C., Hartley J.M., Frisch G., Jenkin G.R.T., Abbott A.P. (2019). Direct extraction of copper from copper sulfide minerals using deep eutectic solvents. Green Chem..

[17] Abbott A.P., Al-Bassam A.Z.M., Goddard A., Harris R.C., Jenkin G.R.T., Nisbet F.J., Wieland M. (2017). Dissolution of pyrite and other Fe–S–As minerals using deep eutectic solvents. Green Chem..

[18] Abbott A.P., Bevan F., Baeuerle M., Harris R.C., Jenkin G.R.T. (2017). Paint casting: A facile method of studying mineral electrochemistry. Electrochem. Commun..

[19] Sánchez L.G., Espel J.R., Onink F., Meindersma G.W., de Haan A.B. (2009). Density, Viscosity, and Surface Tension of Synthesis Grade Imidazolium, Pyridinium, and Pyrrolidinium Based Room Temperature Ionic Liquids. J. Chem. Eng. Data.

[20] Mjalli F.S., Mousa H. (2017). Viscosity of aqueous ionic liquids analogues as a function of water content and temperature. Chin. J. Chem. Eng..

[21] Protsenko V.S., Kityk A.A., Shaiderov D.A., Danilov F.I. (2015). Effect of water content on physicochemical properties and electrochemical behavior of ionic liquids containing choline chloride, ethylene glycol and hydrated nickel chloride. J. Mol. Liq..

[22] Al-Murshedi A.Y.M., Hartley J.M., Abbott A.P., Ryder K.S. (2019). Effect of water on the electrodeposition of copper on nickel in deep eutectic solvents. Trans. IMF.

[23] Du C., Yang H., Chen X.-B., Wang L., Dong H., Ning Y., Lai Y., Jia J., Zhao B. (2018). Effect of coordinated water of hexahydrate on nickel platings from choline–urea ionic liquid. J Mater Sci.

[24] Hartley J.M., Scott S., Rivera R.M., Hunt P., Lucio A.J., Bird P., Harris R., Jenkin G.R.T., Abbott A.P. (2023). Tailoring lixiviant properties to optimise selectivity in E-waste recycling. RSC Sustain..

[25] Jacobson B., Li S., Marin Rivera R., Daly P., Elgar C.E., Mulvihill D.M., Abbott A.P., Feeney A., Prentice P. (2023). A mechanistic study identifying improved technology critical metal delamination from printed circuit boards at lower power sonications in a deep eutectic solvent. Ultrasonics Sonochemistry 101.

[26] Elgar C.E., Ravenhill S., Hunt P., Jacobson B., Feeney A., Prentice P., Ryder K.S., Abbott A.P. (2024). Using ultrasound to increase copper and nickel dissolution and prevent passivation using concentrated ionic fluid. Electrochimica Acta 476.

[27] Slama R.B.H., Gilles B., Chiekh M.B., Bera J.C. (2019). Characterization of focused-ultrasound-induced acoustic streaming. Exp. Therm Fluid Sci..

[28] Klíma J., Bernard C., Degrand C. (1994). Sonoelectrochemistry: Effects of ultrasound on voltammetric measurements at a solid electrode. J. Electroanal. Chem..

[29] Turan M.D., Silva J.P., Sarı Z.A., Nadirov R., Toro N. (2022). Dissolution of Chalcopyrite in Presence of Chelating Agent and Hydrogen Peroxide. Trans Indian Inst Met.

[30] Wang J., Faraji F., Ghahreman A. (2020). Effect of Ultrasound on the Oxidative Copper Leaching from Chalcopyrite in Acidic Ferric Sulfate Media. Minerals.

[31] Larrabure G., Chero-Osorio S., Silva-Quiñones D., Benndorf C., Williams M., Gao F., Gamarra C., Alarcón A., Segura C., Teplyakov A., Rodriguez-Reyes J.C.F. (2021). Surface processes at a polymetallic (Mn-Fe-Pb) sulfide subject to cyanide leaching under sonication conditions and with an alkaline pretreatment: Understanding differences in silver extraction with X-ray photoelectron spectroscopy (XPS). Hydrometall..

[32] Behera S.S., Panda S.K., Mandal D., Parhi P.K. (2019). Ultrasound and Microwave assisted leaching of neodymium from waste magnet using organic solvent. Hydrometall..

[33] Ning P., Meng Q., Dong P., Duan J., Xu M., Lin Y., Zhang Y. (2020). Recycling of cathode material from spent lithium ion batteries using an ultrasound-assisted DL-malic acid leaching system. Waste Manag..

[34] Jiang F., Chen Y., Ju S., Zhu Q., Zhang L., Peng J., Wang X., Miller J.D. (2018). Ultrasound-assisted leaching of cobalt and lithium from spent lithium-ion batteries. Ultrason. Sonochem..

[35] Xuan W., Chagnes A., Xiao X., Olsson R.T., Forsberg K. (2022). Antisolvent Precipitation for Metal Recovery from Citric Acid Solution in Recycling of NMC Cathode Materials. Metals.

[36] Zhang X., Wang J., Chen J., Lyu B., Yuan J. (2024). Material Removal and Surface Modification of Copper under Ultrasonic-Assisted Electrochemical Polishing. Processes.

[37] Li L., King A., Davis K., Yu B. (2023). Electrochemical Kinetics Study of Ultrasound-Assisted Chalcopyrite Oxidation. J. Sustain. Metall..

[38] Kang J., Shin Y., Tak Y. (2005). Growth of etch pits formed during sonoelectrochemical etching of aluminum. Electrochim. Acta.

[39] Daskalopoulou E., Hunt P., Elgar C.E., Yang M., Abbott A.P., Hartley J.M. (2024). Overcoming passivation through improved mass transport in dense ionic fluids. Faraday Discuss..

[40] Kumari A., Dipali N.S., Randhawa S.K.S.h. (2021). Electrochemical treatment of spent NdFeB magnet in organic acid for recovery of rare earths and other metal values. Journal of Cleaner Production 309.

[41] Zakotnik M., Harris I.R., Williams A.J. (2009). Multiple recycling of NdFeB-type sintered magnets. J. Alloy. Compd..

[42] Murr L.E., Lerner S.L. (1977). Transmission electron microscopic study of defect structure in natural chalcopyrite (CuFeS2). J Mater Sci.

[43] M.A. Rabeea, C.E. Elgar, J.M. Hartley, A. Walton, A.P. Abbott, J.M. Yang, Selective, ultrasound-assisted electrochemical recovery of rare-earth elements from end-of-life neodymium magnet, Submitted. (2025).

[44] Ahmed E.I., Ryder K.S., Abbott A.P. (2021). Corrosion of iron, nickel and aluminium in deep eutectic solvents. Electrochim. Acta.

[45] Abbott A.P., Frisch G., Hartley J., Karim W.O., Ryder K.S. (2015). Anodic dissolution of metals in ionic liquids. Prog. Nat. Sci.: Mater. Int..

[46] Pateli I.M., Abbott A.P., Jenkin G.R.T., Hartley J.M. (2020). Electrochemical oxidation as alternative for dissolution of metal oxides in deep eutectic solvents. Green Chem..

[47] A.P. Abbott, N. Dsouza, P. Withey, K.S. Ryder*, Electrolytic processing of superalloy aerospace castings using choline chloride-based ionic liquids, Transactions of the IMF 90 (2012) 9–14. doi: 10.1179/174591912X13228247936644.

[48] Boschen R.E., Rowden A.A., Clark M.R., Gardner J.P.A. (2013). Mining of deep-sea seafloor massive sulfides: A review of the deposits, their benthic communities, impacts from mining, regulatory frameworks and management strategies. Ocean Coast. Manag..

[49] Martínez-Gómez V.J., Fuentes-Aceituno J.C., Pérez-Garibay R., Lee J. (2018). A study of the electro-assisted reductive leaching of a chalcopyrite concentrate in HCl solutions. Part i: Kinetic Behavior and Nature of the Chalcopyrite Reduction, Hydrometallurgy.

[50] Lu Z.Y., Jeffrey M.I., Lawson F. (2000). An electrochemical study of the effect of chloride ions on the dissolution of chalcopyrite in acidic solutions. Hydrometall..

[51] Bevan F., Galeb H., Black A., Pateli I.M., Allen J., Perez M., Feldmann J., Harris R., Jenkin G., Abbott A., Hartley J. (2021). A Unified Method for the Recovery of Metals from Chalcogenides. ACS Sustainable Chem. Eng..

[52] Sun G., Jiang M., Wang S., Fu L., Zuo Y., Zhang G., Hu Z., Zhang L. (2023). Enhancement of copper metal dissolution in sulfuric acid solution with oxygen and ultrasound. J. Mater. Res. Technol..

