# Supplementary material for: Electrochemical etching of metals and minerals using ultrasound in deep eutectic solvents
Source: Ultrason Sonochem. 2025 May 27;119:107403. doi: 10.1016/j.ultsonch.2025.107403 (PMC12159910; doi:10.1016/j.ultsonch.2025.107403)
Supplement: Supplementary Data 1 [file mmc1.docx]

**Electrochemical etching of metals and minerals using ultrasound in deep eutectic solvents**

Philip Hunt,^a^ Jennifer M. Hartley,^a^ Muwafaq A. Rabeea,^a,b^ Andrew P. Abbott,^a^ Christopher E. Elgar^a^*

*^a^ School of Chemistry, University of Leicester, Leicester, LE1 7RH*

*^b^ Department of Applied Chemistry, College of Applied Sciences-Hit, University Of Anbar, Iraq*

* Corresponding author: [cee17@leicester.ac.uk](mailto:cee17@leicester.ac.uk)

# Supplementary information


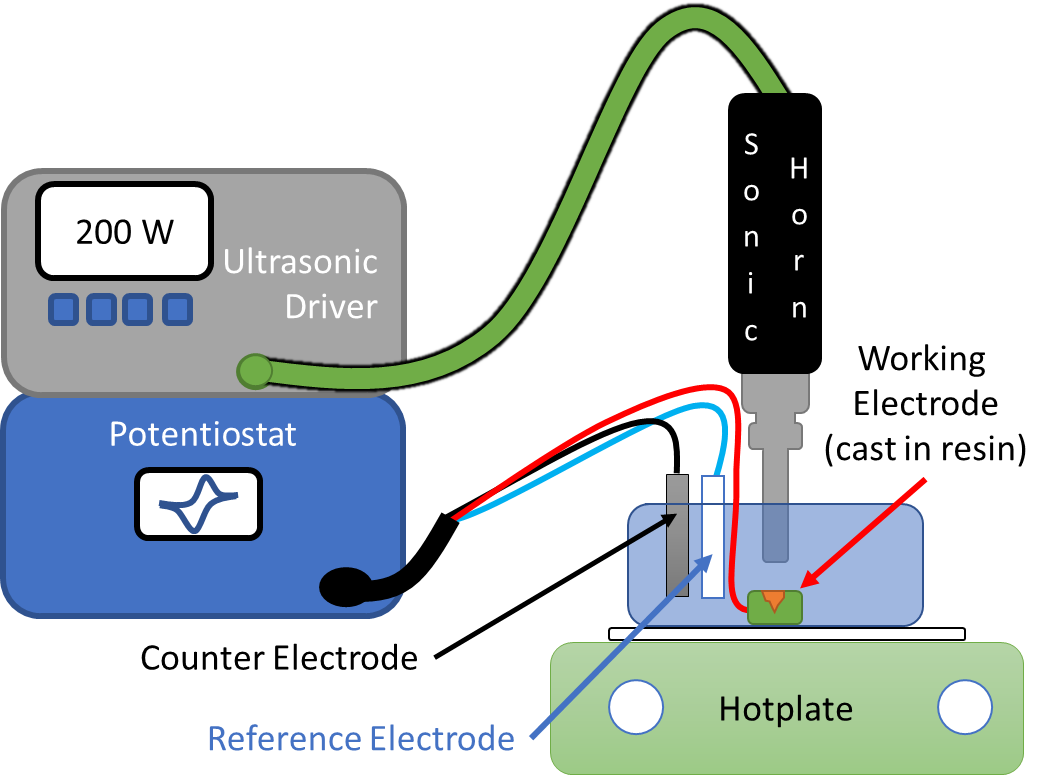


**Figure S1**: Experimental set up for sonoelectrochemical experiments.

| 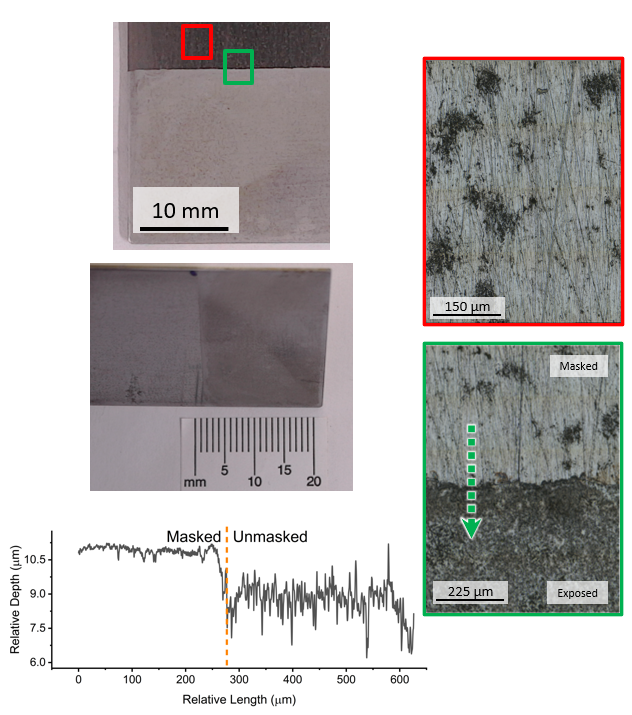 | 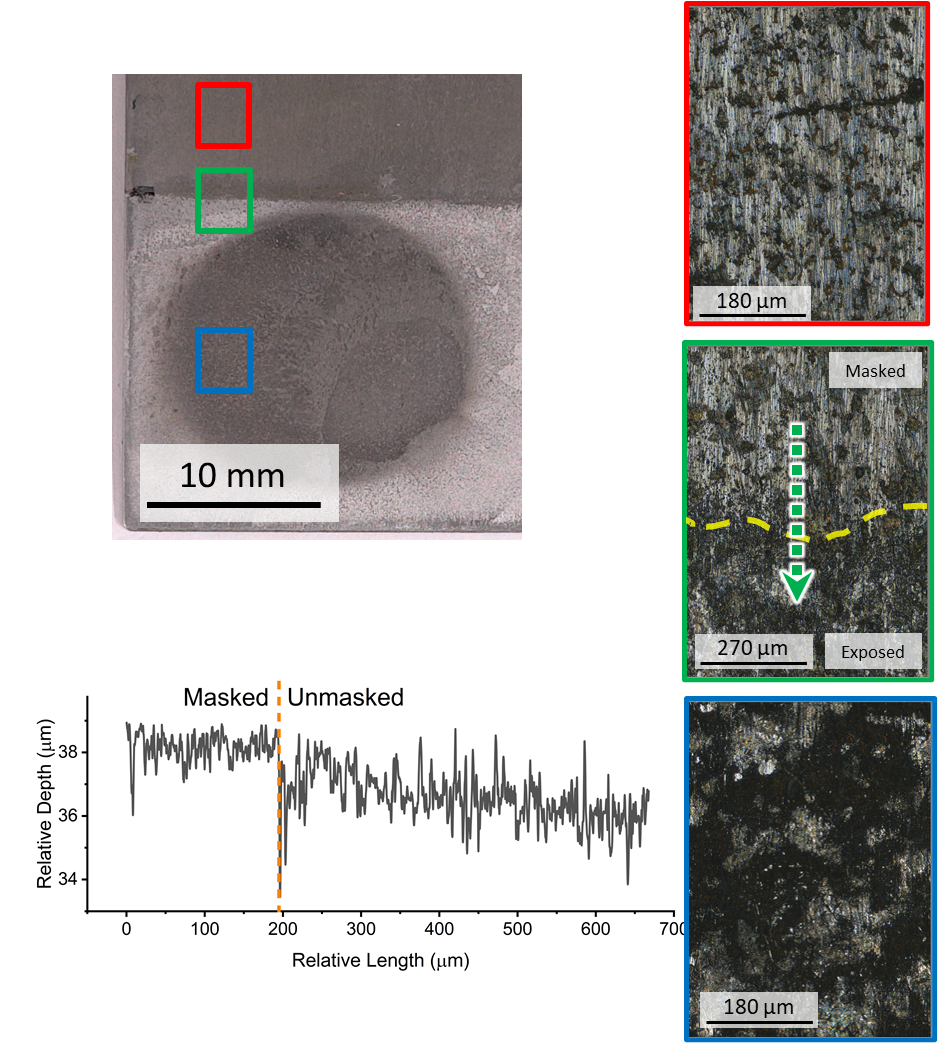 |
| --- | --- |
| **Figure S2:** Photographs of mild steel etched under left: silent, and right: ultrasonic conditions. Electrochemical etching was carried out at a constant applied current density of 40 mA cm^‒2^, for 2 min, at a constant temperature of 50 °C. The applied ultrasound was 132 W cm^‒2^. Dashed green lines indicate where the surface height lines were measured. Graphs show the change in surface height, and give the reader an indication of change in surface roughness between masked and unmasked. | |

| 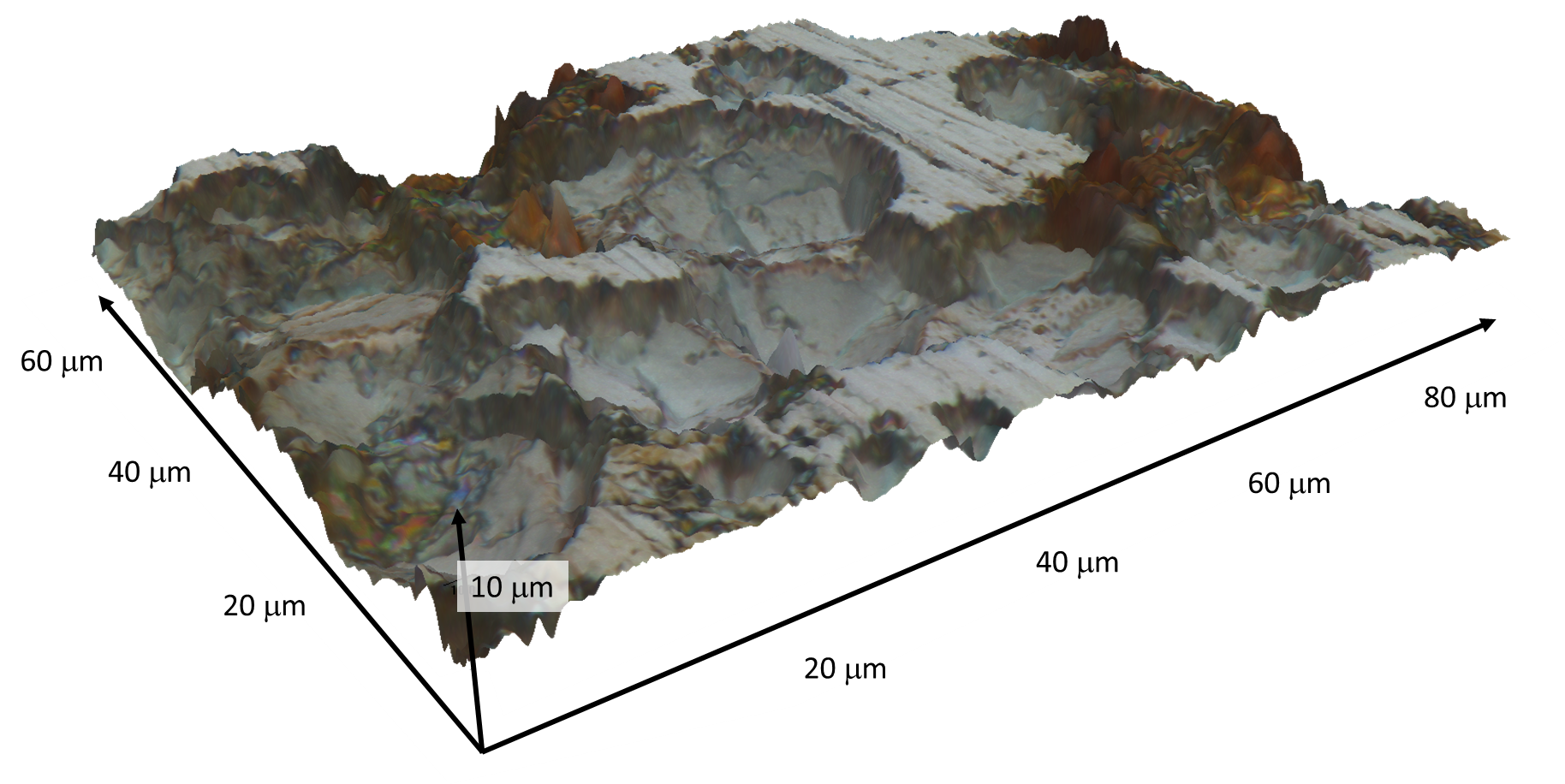 |
| --- |
| **Figure S3:** 3D optical topography image of mild steel post sonication electrochemistry experiment. |

| (a) | (b) |
| --- | --- |
| 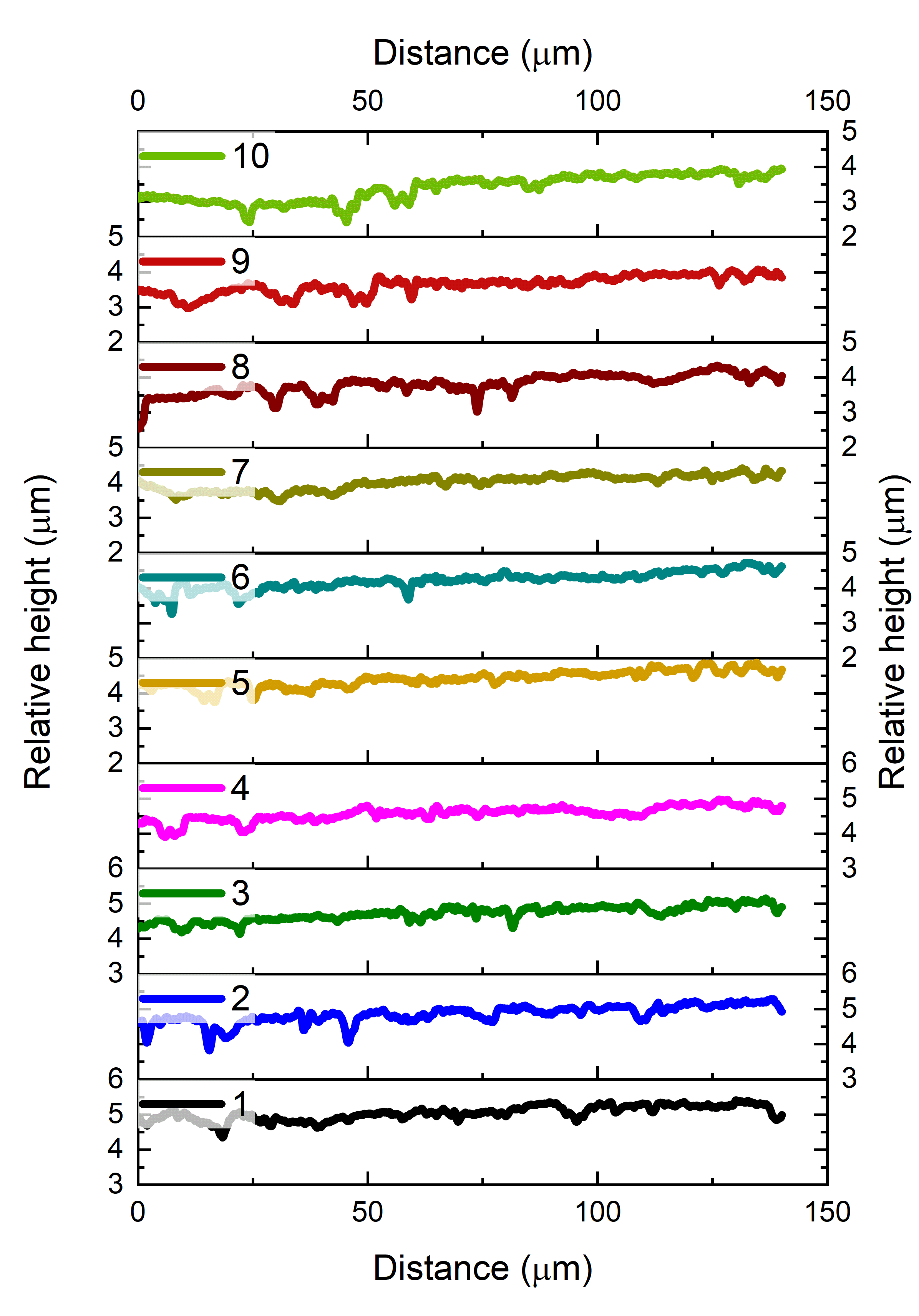 | 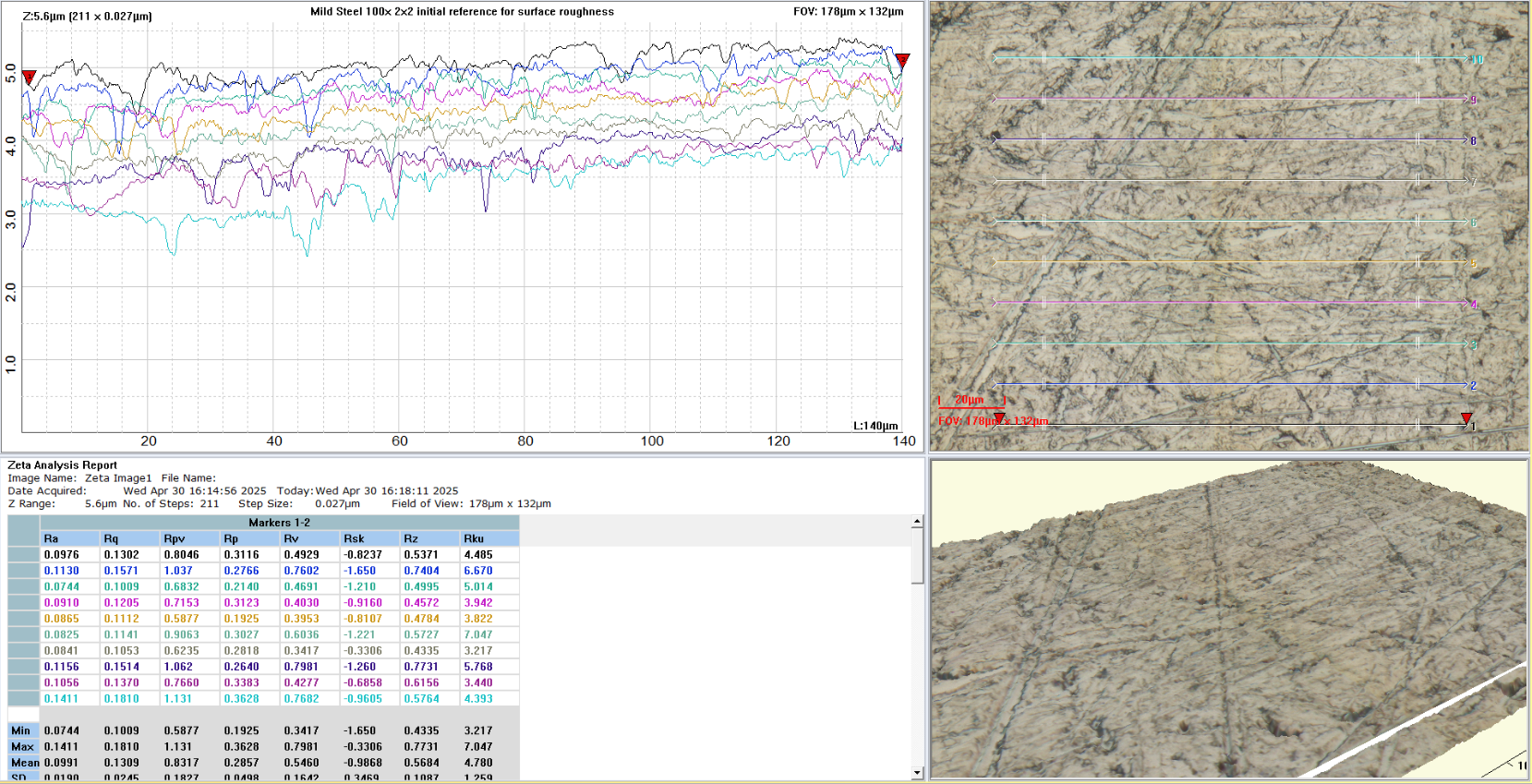 |
| (c) | (d) |
| 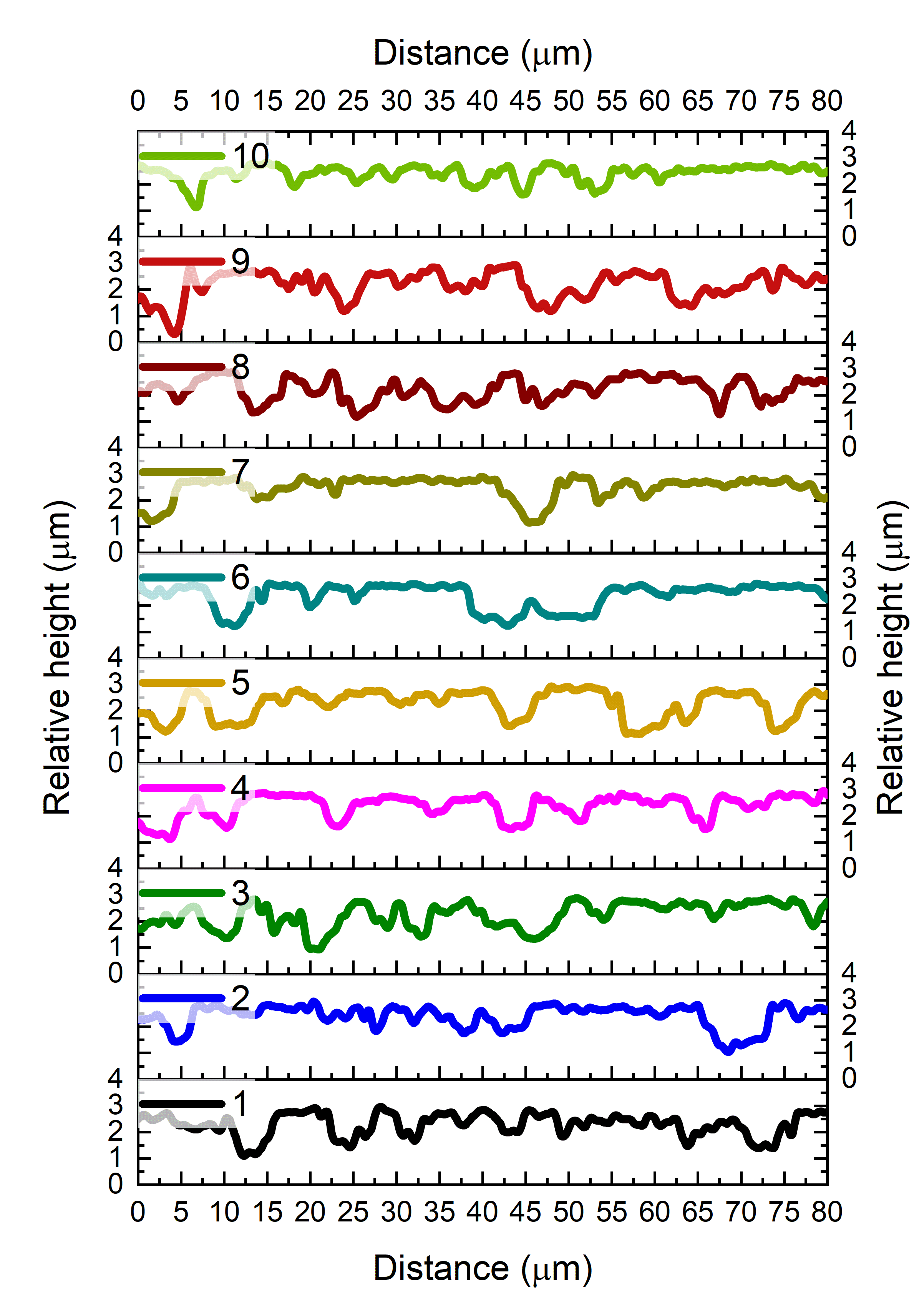 | 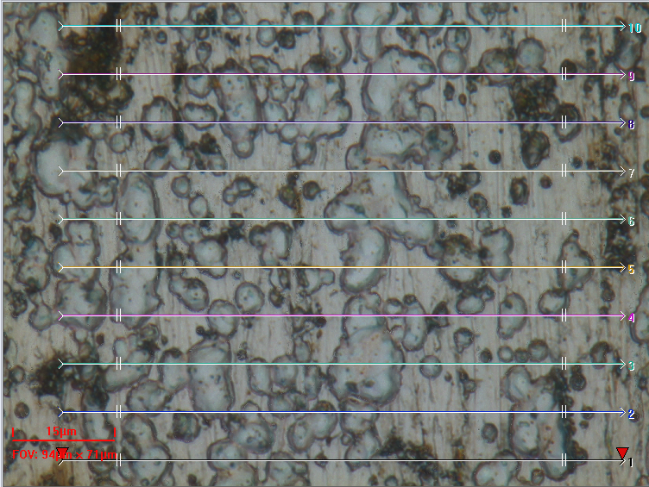 |
| (e) | (f) |
| 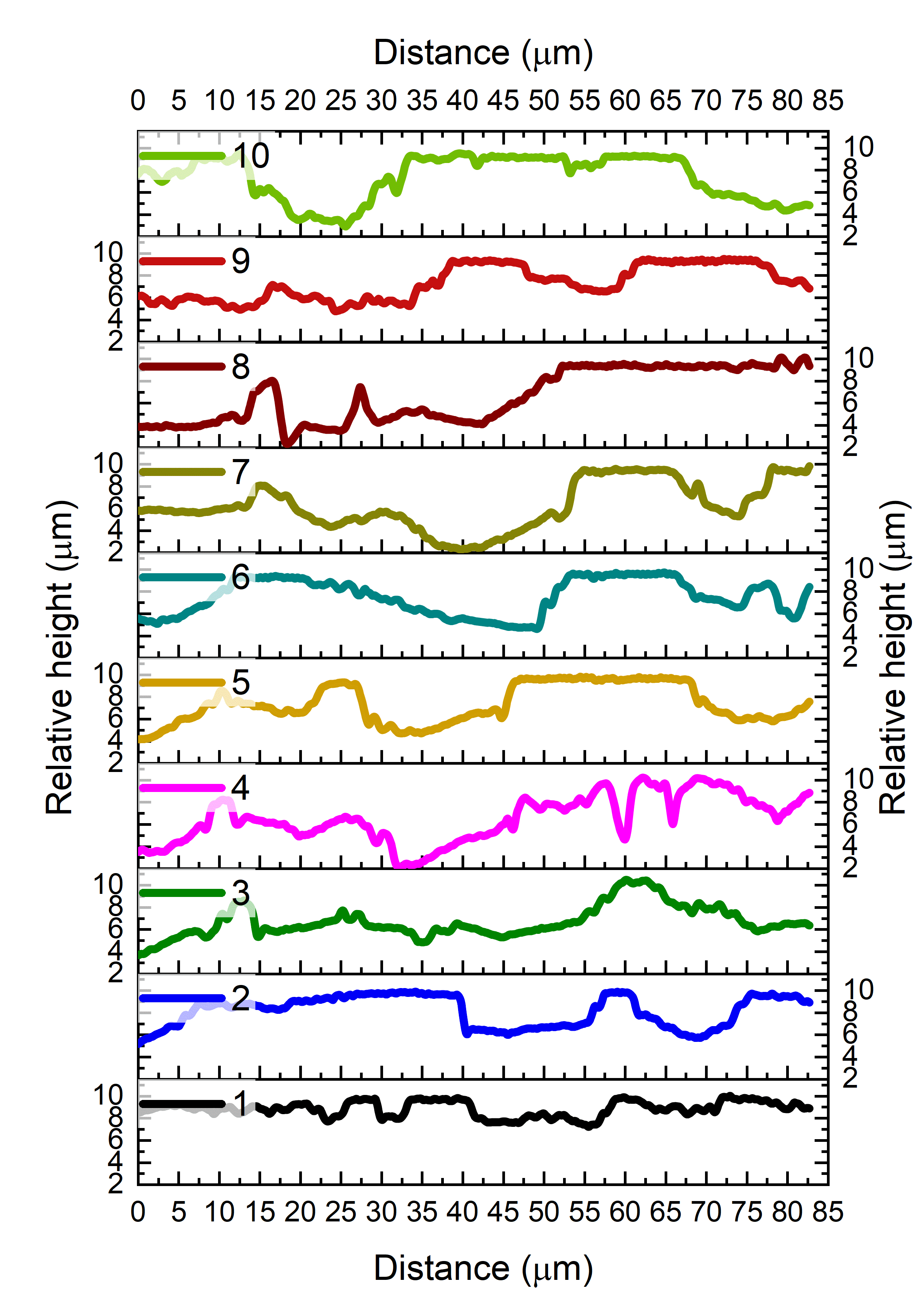 | 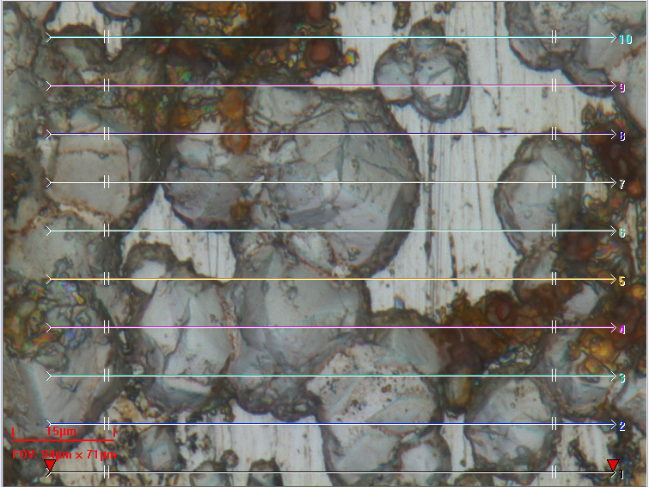 |
| **Figure S4:** Surface roughness profiles used to calculate average Ra values on Pre-exposure (a)+(b), Silent electrochemical etch (c) + (d) and after sonoelectrochemical etch (e)+(f). Roughness was calculated using the inbuilt functions on the Zeta3D software. | |

| **a)** |
| --- |
| 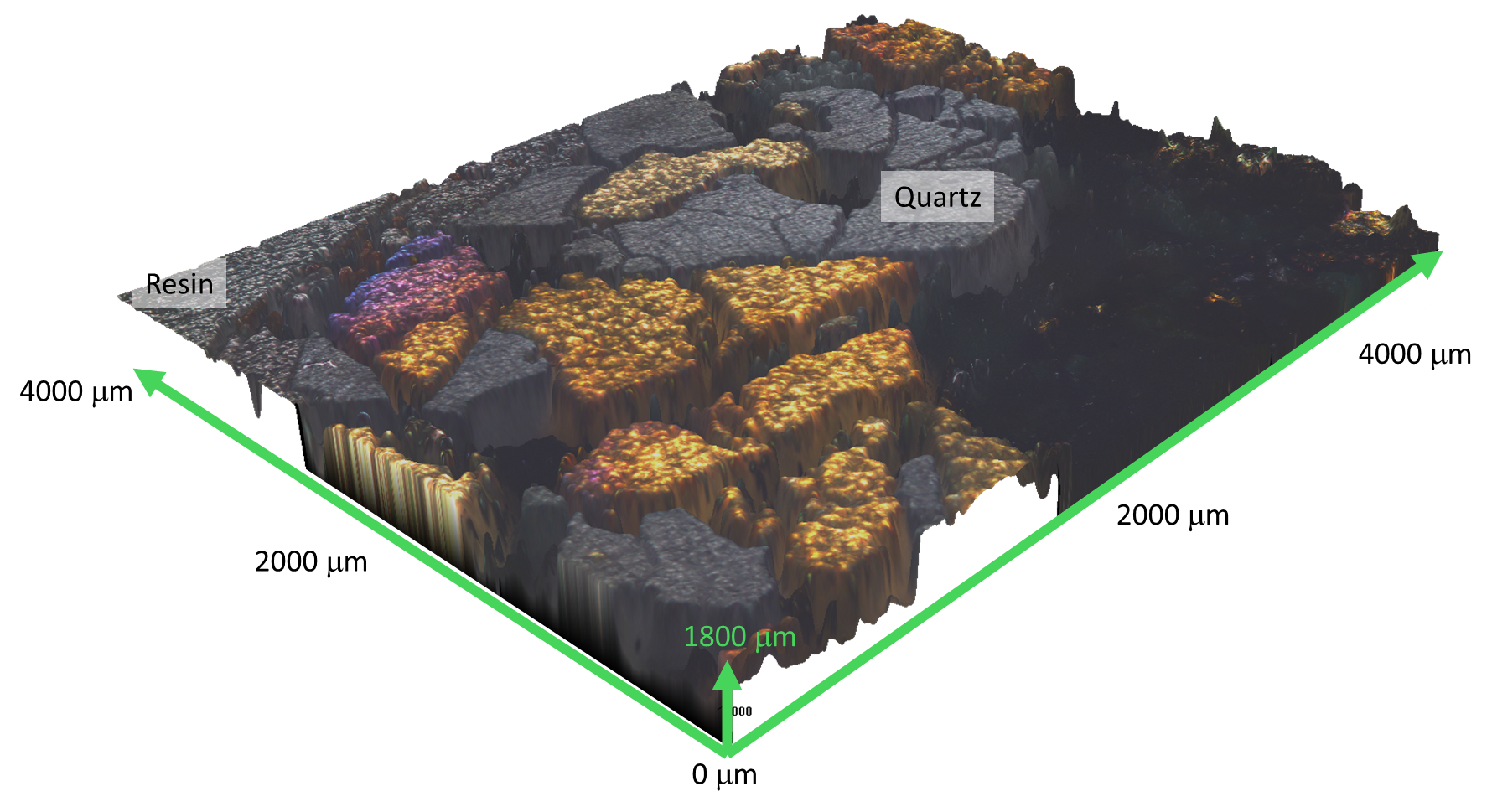 |
| **b)** |
| 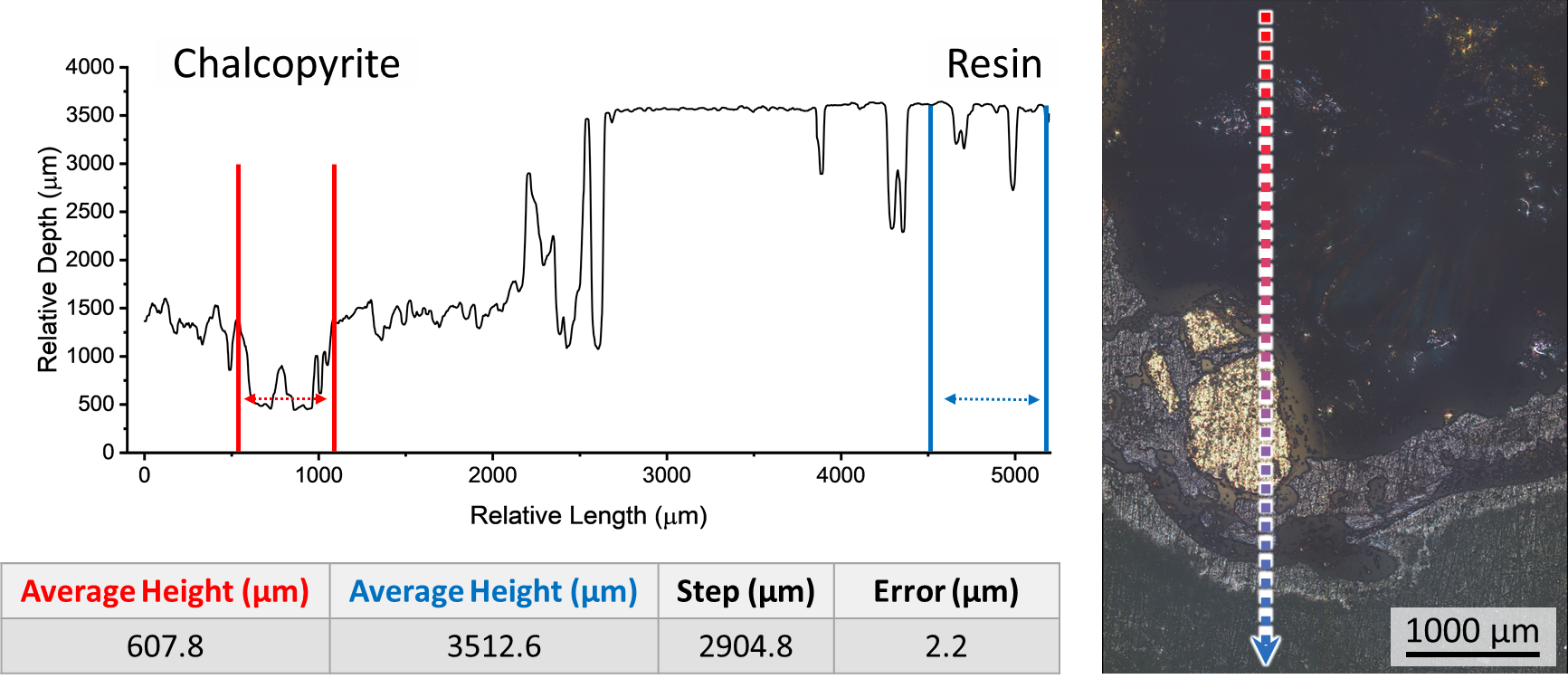 |
| **Figure S5:** a) 3D optical microscopy image of the surface of the sonoelectrochemically etched chalcopyrite taken at 5x on Zeta 20 microscope. Electrochemical etching was carried out at a constant applied current density of ca. 5 mA cm^‒2^, for 2 minutes, at a constant temperature of 50 °C. The applied ultrasound was 132 W cm^‒2^. b) Same sample showing the cross sectional depth across the area with the most removed material. |

| 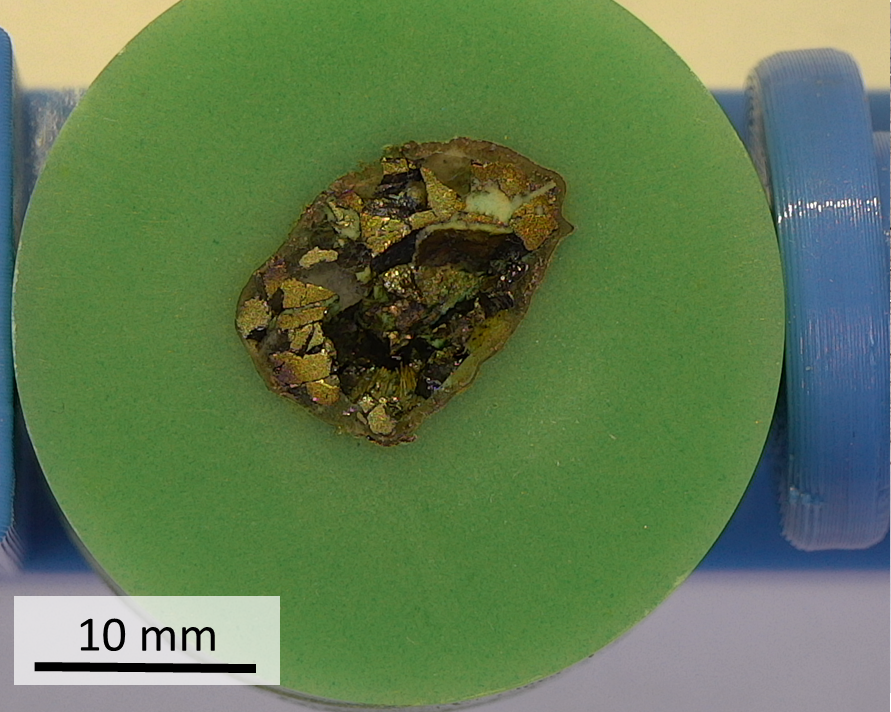 |
| --- |
| **Figure S6** Optical image of chalcopyrite exposed to a further 2 minutes of sonochemical dissolution, with no applied potential in pure ChCl:2EG solution, after having previously undergone sonoelectrochemical etching. The applied ultrasound was 132 W cm^‒2^ at a constant temperature of 50 °C. |

| 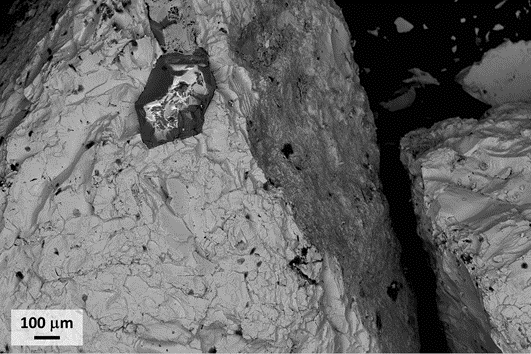 | |  |
| --- | --- | --- |
| 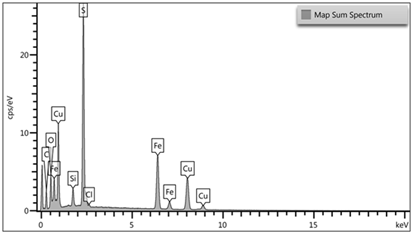 | 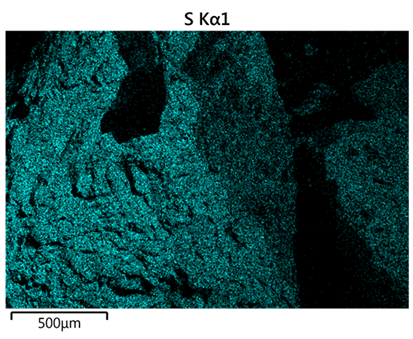 |  |
| 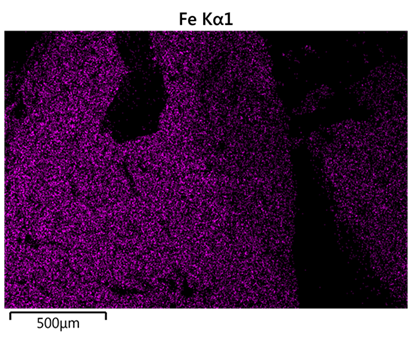 | 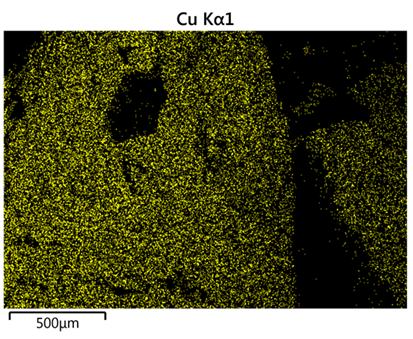 |  |
| 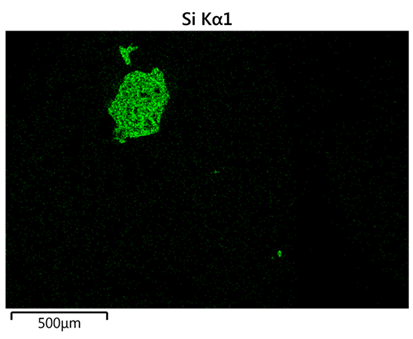 | 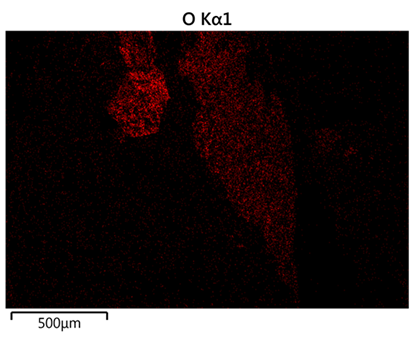 |  |
| **Figure S7:** SEM EDX maps for large pieces of chalcopyrite removed during sonoelectrochemical experiments. | |  |
| 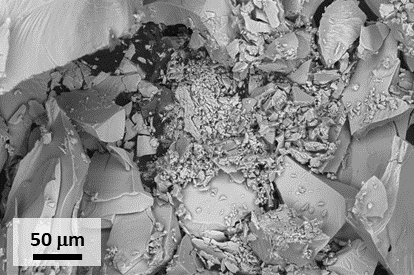 | 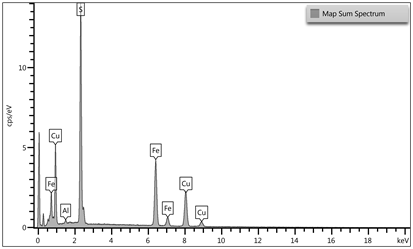 | |
| 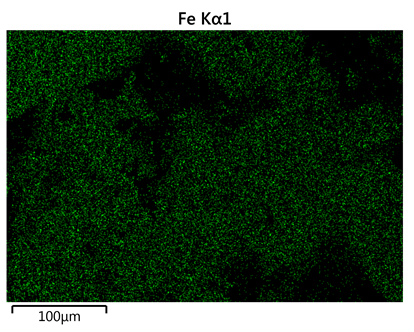 | 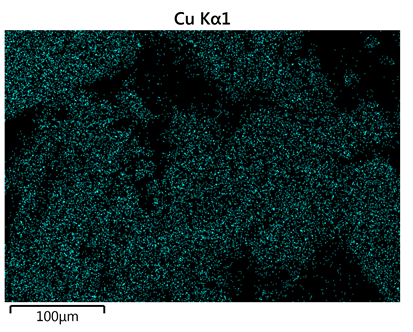 | |
| 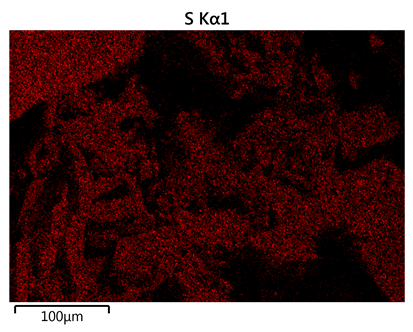 | 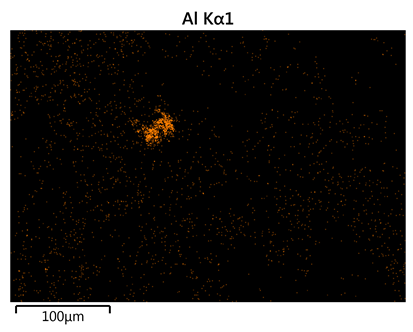 | |
| **Figure S8:** SEM EDX maps of the smaller pieces of chalcopyrite removed during sonoelectrochemical experiments. | | |

| 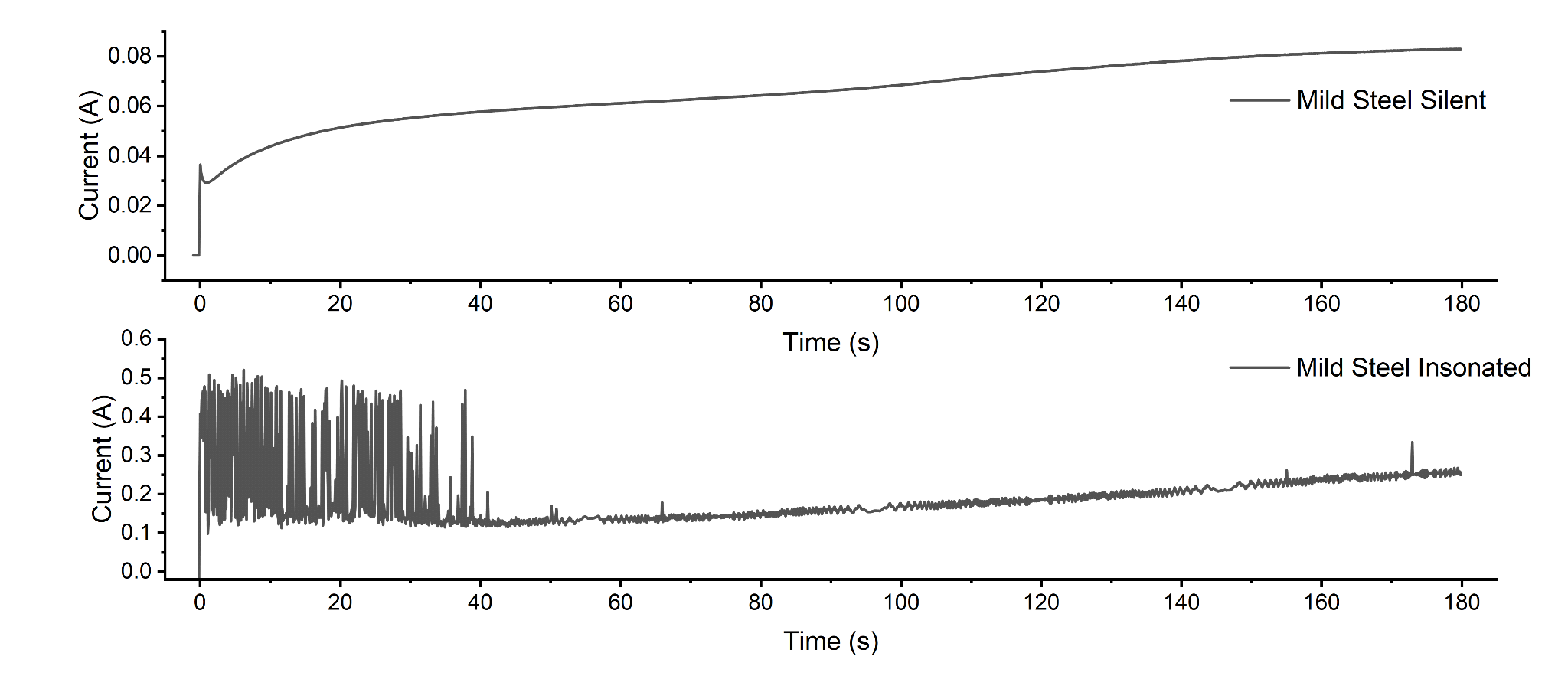 |
| --- |
| **Figure S9:** Chronoamperometry traces for the mild steel plates under silent and insonated conditions. In both cases the samples were held at +1.5 V versus 0.09 mol dm^‒3^ AgCl/Ag in ChCl:2EG reference. Ultrasound was applied to the insonated sample at 132 W cm^-2^ over the full length of the CA scan. |
